# Vacuoles, E1 Enzyme, X-linked, Autoinflammatory, and Somatic (VEXAS) Syndrome: A Diagnostic and Therapeutic Conundrum

**DOI:** 10.7759/cureus.79338

**Published:** 2025-02-19

**Authors:** Sofia Miranda, Carolina Roias, Joana Rua, Fernando Salvador

**Affiliations:** 1 Internal Medicine, Hospital do Divino Espírito Santo, Ponta Delgada, PRT; 2 Internal Medicine, Unidade Local de Saúde de Trás-os-Montes e Alto Douro, Vila Real, PRT

**Keywords:** il-6 antagonist, inflammation, jak inhibitor, myelodysplastic syndrome, vexas syndrome

## Abstract

Vacuoles, E1 enzyme, X-linked, autoinflammatory, and somatic (VEXAS) syndrome is an adult-onset systemic autoinflammatory condition notable for being simultaneously an autoinflammatory syndrome and a hematologic disease. Here, we present a clinical case of a 67-year-old man referred to the Autoimmune Diseases Outpatient Clinic for a two-year history of maculopapular rash involving the face, trunk, back, and upper limbs. At the time, the patient had already undergone three skin biopsies, with conflicting results. His physical exam was only remarkable for multiple roundly shaped maculopapules involving the trunk, back, and extremities. Laboratory evaluation revealed elevated erythrocyte sedimentation rate and C-reactive protein, increased alpha-2 zone in the serum protein electrophoresis, and positive IgM antibodies against *Borrelia burgdorferi*. A diagnosis of Lyme disease was assumed, and a trial of doxycycline for two weeks was introduced, as well as hydroxychloroquine at a maximum dosage of 5 mg/kg, without noticeable improvement. A new skin biopsy was performed, suggestive of amyopathic dermatomyositis. Prednisolone at a dosage of 0.5 mg/kg/day was then started, with some improvement. Two years later, the patient developed two episodes of deep vein thrombosis along with a de novo macrocytic anemia and neutropenia. A positron emission computed tomography showed nonspecific medullar activation, whereas bone marrow aspirate and biopsy suggested a diagnosis of myelodysplastic syndrome. The patient became progressively more reliant on regular blood product transfusions, and, three years later, a recrudescence of his skin lesions was also noted under a prednisolone dosage of 5 mg/day. Although an increase in prednisolone to 0.5 mg/kg/day and an association with methotrexate and dapsone were tried, no improvement was noted. Bone marrow aspirate and biopsy were repeated, with aberrant vacuolized bone marrow cells being observed. A genetic analysis revealed a Met41Leu mutation at the ubiquitin-activating enzyme 1 (UBA1) gene, confirming the diagnosis of VEXAS syndrome. The patient was started on tocilizumab 6 mg/kg, but a profound neutropenia required a therapeutical switch to ruxolitinib.

## Introduction

Vacuoles, E1 enzyme, X-linked, autoinflammatory, and somatic (VEXAS) syndrome is an adult-onset systemic autoinflammatory condition notable for being simultaneously an autoinflammatory syndrome and a hematologic disease [1]. A study by Beck et al. estimated a prevalence of one in 13591 adults as of 2023, affecting mainly men over the age of 50 [2]. However, this prevalence might be underestimated, as its recent identification, protean manifestations, and significant overlap with other more common diseases can make the diagnosis more challenging.

Whereas monogenic autoinflammatory conditions have been classically identified through a "top-down" approach, VEXAS syndrome was firstly identified through a whole genome sequencing of more than 2000 patients, during which a mutation in the methionine-41 residue of ubiquitin-activating enzyme 1 (UBA1) was found [3]. Since then, more mutations have been found, with mutation c.121A>G (p.Met41Val) being associated with the worse prognosis [4]. Besides its manner of discovery, VEXAS syndrome is also remarkable due to the fact that it stems from somatic mutations. Indeed, most autoinflammatory conditions are Mendelian disorders, while VEXAS arises from somatic mutations acquired later in life [5].

The two largest cohorts, in the United States/United Kingdom and in France, provided great insights regarding the heterogeneity and frequency of the clinical features of this highly variable syndrome [5,6]. Skin lesions (including neutrophilic dermatoses, vasculitic rashes, or urticaria) were the most common manifestation. Noninfectious fever, constitutional symptoms, lung involvement (including pulmonary infiltrates or pleural effusions), arthralgia/arthritis, relapsing chondritis, ocular symptoms, venous thrombosis, and lymphadenopathy were the next most frequent features [5,6]. However, as a true multisystemic disease, almost all organs and systems seemed to be affected, with reports of myocarditis or orchitis also being noted in these cohorts. Although guidelines and diagnostic criteria are still elusive, some red flags have been proposed, such as age >50 years, male sex, systemic inflammation, multiorgan involvement, myelodysplastic syndrome, thrombocytopenia, nasal and/or auricular chondritis, skin lesions, pulmonary involvement, and deep vein thrombosis [7].

Due to a high mortality rate, VEXAS syndrome requires a timely diagnosis and aggressive treatment strategy [8]. There are currently two established strategies: to reduce the activity of the mutated clone, employing the usage of hypomethylating agents or advancing to allogeneic stem cell transplantation (typically in severe cases), or to inhibit the cytokine storm using glucocorticoids, anti-interleukin-6 agents, or Janus kinase (JAK) inhibitors [5]. Furthermore, symptomatic therapy, such as blood transfusions and infection prophylaxis, is also frequently considered. Other therapies have also been tried, like anti-interleukin 1 and anti-tumor necrosis factor alpha agents, but mainly in case reports, and more evidence is necessary to prove their benefits [9].

This article was previously presented as a meeting abstract at the X Congresso Nacional de Autoimunidade/XXIX Reunião Anual do NEDAI on June 29, 2024.

## Case presentation

A 67-year-old man was initially referred to the Autoimmune Diseases Outpatient Clinic for a two-year history of maculopapular rash involving the face, trunk, back, and upper limbs. His past medical history included primary hypertension, dyslipidemia, and benign prostatic hyperplasia. His chronic medication was bisoprolol 5 mg/day, simvastatin 10 mg/day, silodosin 8 mg/day, and *Serenoa repens*.

**Figure 1:**
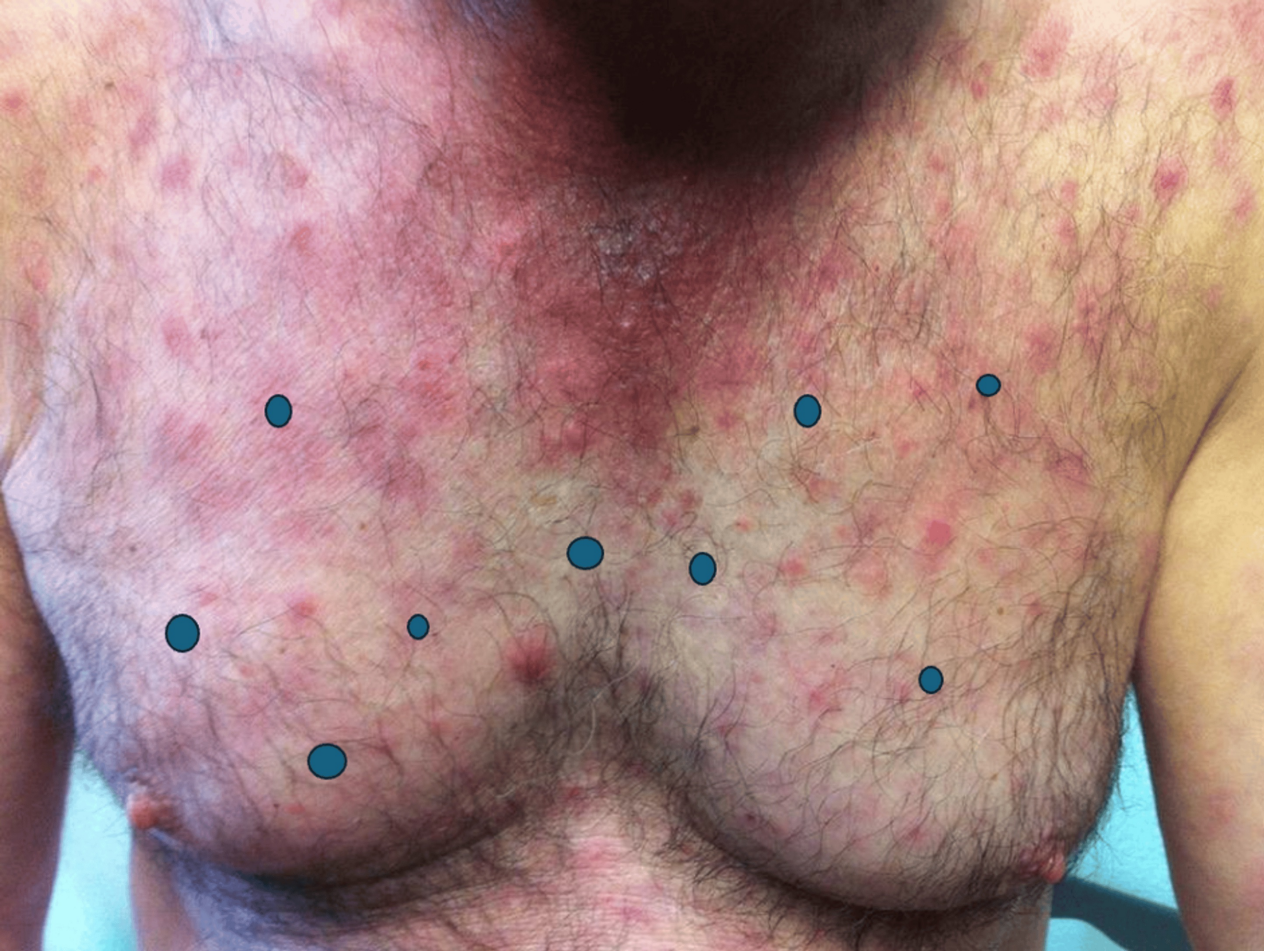
Skin lesions at the initial consultation

**Figure 2:**
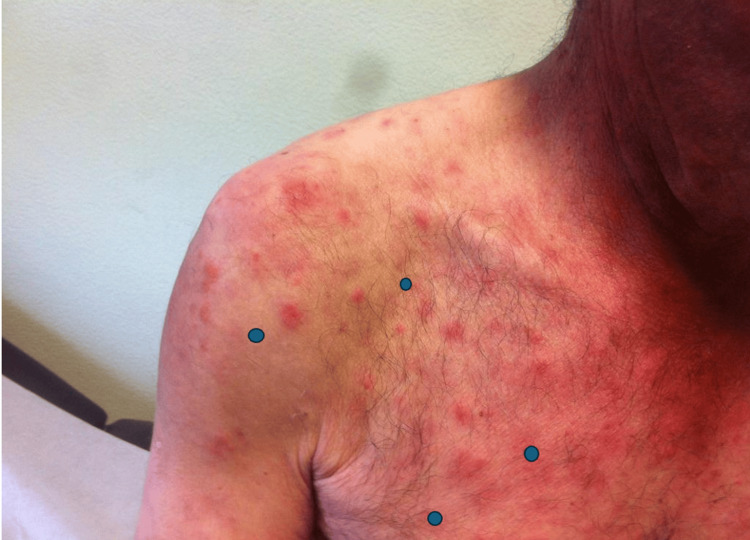
Skin lesions at the initial consultation

At the time, the patient had already undergone three skin biopsies, with conflicting results: the first one was inconclusive, the second one was compatible with Lyme disease (no polymerase chain reaction test was performed), and the last one was compatible with morphea. His physical exam was only remarkable for multiple roundly shaped maculopapules involving the trunk, back, and extremities (Figure 1 and Figure 2). 

Laboratory evaluation revealed a raised erythrocyte sedimentation rate (ESR) (50 mm/h; range: <30 mm/h) and C-reactive protein (CRP) (2 mg/dL; range: <1 mg/dL). Comprehensive serological evaluations for systemic autoimmune disorders were unremarkable, including negative rheumatoid factor, antinuclear antibodies (ANA), antineutrophil cytoplasmic antibodies (ANCA), anti-double-stranded deoxyribonucleic acid (anti-dsDNA), anti-extractable nuclear antigen (anti-ENA), and immunoblot for systemic sclerosis and normal levels of C3 and C4. Serum protein electrophoresis showed increased alpha-2 zone (0.12 g/dL; range: <0.1 g/dL). IgM antibodies against* Borrelia burgdorferi* were positive, while IgG antibodies were inconclusive. Imaging (thoracic, abdominal, and pelvic computed tomography (CT)) showed no evidence of solid tumors, whereas endoscopic exams revealed no suspicious lesions. Although serology was equivocal due to the possibility IgM might constitute a false positive and IgG was inconclusive (a chronic presentation is typically associated with its positivity), a working diagnosis of Lyme disease/chronic was assumed, and a trial of doxycycline for two weeks was introduced, as well as hydroxychloroquine at a maximum dosage of 5 mg/kg due to its known effects on skin lesions of various nature. 

However, there was no noticeable improvement, and, after a referral to the Dermatology Outpatient Clinic, a new skin biopsy was performed. This time the findings were suggestive of amyopathic dermatomyositis. Both creatine kinase and aldolase levels were normal, and an immunoblot for idiopathic inflammatory myositis was negative. Prednisolone at a dosage of 0.5 mg/kg/day was started, with some improvement.

For almost two years, the patient remained stable without specific treatment besides corticosteroids (follow-up every three to four months). However, he presented with two episodes of deep vein thrombosis in the same year (both in his legs and one of them associated with pulmonary embolism) along with a de novo and progressive macrocytic anemia and neutropenia (no vitamin deficiencies were noted). Anticoagulation was introduced and an extensive study was again performed. Laboratory studies showed a raised rheumatoid factor, but ANA, ANCA, anti-ENA, anti-dsDNA, and antiphospholipid antibodies remained negative. A whole-body CT and endoscopic exams still didn't reveal any significant findings. A positron emission CT showed nonspecific medullar activation. Bone marrow aspirate and biopsy were suggestive of a diagnosis of myelodysplastic syndrome with a normal karyotype and low Revised International Prognostic Scoring System (IPSS-R). No vacuoles were detected. 

The patient became progressively more reliant on regular blood product transfusions, and, three years later, a recrudescence of his skin lesions was also noted under a prednisolone dosage of 5 mg/day. Even though the dosage was increased to 0.5 mg/kg/day and an association with firstly methotrexate and then dapsone was tried, skin lesions didn't subside. Bone marrow aspirate and biopsy were repeated, with aberrant vacuolized bone marrow cells being observed. Subsequently, and considering the presence of mucocutaneous and hematological manifestations, a clinical suspicion of VEXAS syndrome was raised. Indeed, a genetic analysis revealed a Met41Leu mutation at the UBA1 gene, confirming the diagnosis. 

**Figure 3:**
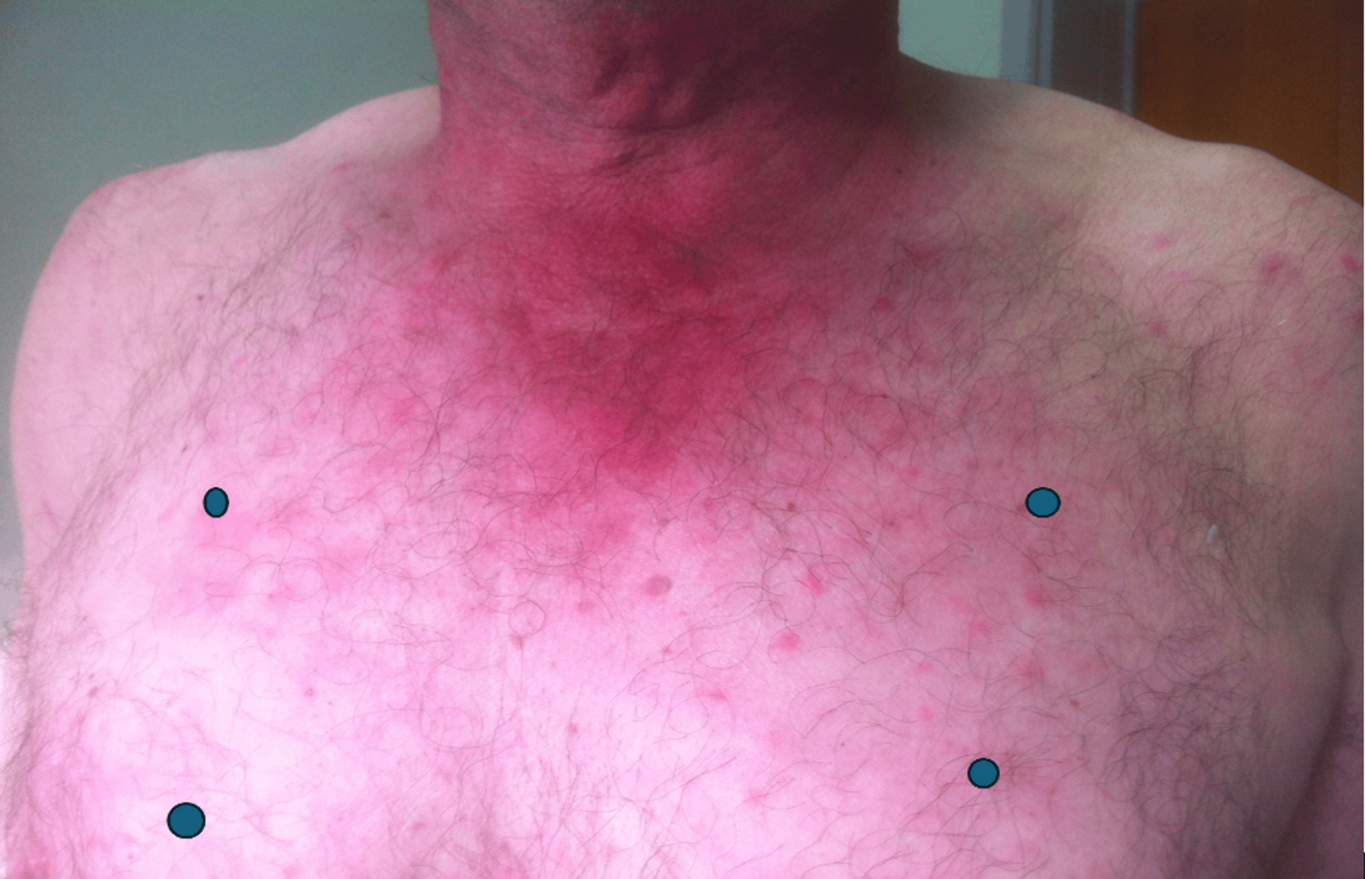
Skin lesions after ruxolitinib

**Figure 4:**
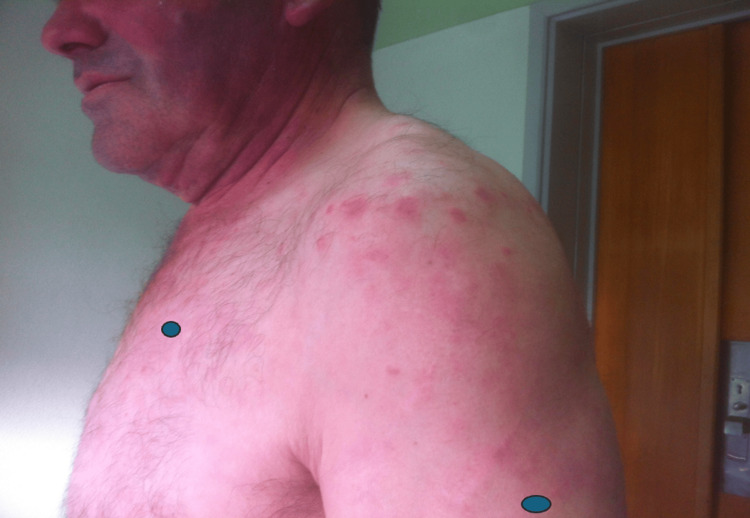
Skin lesions after ruxolitinib

**Figure 5:**
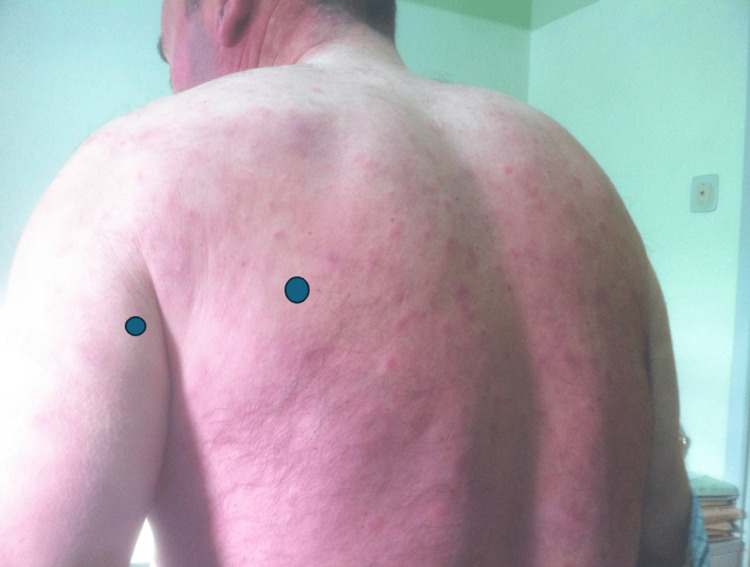
Skin lesions after ruxolitinib

As an inflammatory phenotype seemed more preeminent and the patient had several thrombotic events in the past, he was started on tocilizumab 6 mg/kg. Even though some clinical improvement was observed, a profound neutropenia required a therapeutical switch to ruxolitinib. Since then and for almost two years, not only have the skin lesions improved (Figures 3-5), but the number of blood product transfusions has also decreased. No new thromboembolic events were noted. 

## Discussion

Like most patients with VEXAS syndrome, multiple consultations with several specialists and several exams were necessary before a sound biological explanation was found for our patient. Initially, he presented with cutaneous symptoms and raised inflammatory markers. A study by Hines et al. identified 20 distinct dermatological presentations, with neutrophilic dermatoses being the most frequent [10]. Our patient underwent four biopsies, with diverging results, but no neutrophilic infiltrate was ever detected, making the diagnosis even more challenging.

The patient subsequently developed thrombotic events and myelodysplastic syndrome, which can be considered red flags for VEXAS syndrome, as previously mentioned [7]. These findings were consistent with previous reports indicating a high prevalence of thromboembolic venous events [5,6]. While systemic and vascular inflammation seem to be important contributors, there is a lack of knowledge regarding its pathophysiology. An extensive investigation is still deemed necessary to exclude any underlying malignancy, as thrombosis may indicate an occult cancer. Through two comprehensive diagnostic workups separated by several years, no solid malignancy was ever found. However, the patient developed features of myelodysplastic syndrome, requiring regular blood transfusions although his IPSS-R was low. This score is based on the number and depth of cytopenias and marrow blasts and the presence of cytogenetic abnormalities, but the mutations associated with the development of VEXAS syndrome have not yet been included, which might account for this apparent discrepancy regarding patients' true prognosis [11]. 

A therapeutic strategy based on inhibiting the cytokine storm using glucocorticoids and anti-interleukin-6 agents was firstly pursued, considering that the patient's performance status at the time was already quite deteriorated and contraindicated more aggressive treatments. Although a retrospective study by Heiblig et al. showed JAK inhibitors, specially ruxolitinib, were associated with significantly better clinical and biochemical response [12], the patient's venous thromboembolism history and known association between thromboembolism and JAK inhibitors made the authors choose tocilizumab as the first therapeutic line to be employed. However, the development of profound neutropenia as a side effect made the authors decide to introduce ruxolitinib. A complete blood transfusion independence was not achieved, but its frequency was significantly reduced, bringing more quality of life to our patient. 

## Conclusions

Diagnosing VEXAS syndrome can be laborious due to the heterogeneity of its presentation. On the other hand, its manifestations overlap frequently with other autoimmune, autoinflammatory, or neoplastic diseases, impeding an early diagnosis. Therefore, awareness of this condition and a high level of clinical suspicion are vital to establish a diagnosis. A comprehensive and multidisciplinary approach is advisable and recommended not only to allow an early diagnosis but also to guarantee an adequate therapeutic strategy and follow-up.
